# Self-Reported Screen Time and Cardiometabolic Risk in Obese Dutch Adolescents

**DOI:** 10.1371/journal.pone.0053333

**Published:** 2012-12-28

**Authors:** Teatske M. Altenburg, Geesje H. Hofsteenge, Peter J. M. Weijs, Henriette A. Delemarre-van de Waal, Mai J. M. Chinapaw

**Affiliations:** 1 VU University Medical Center, EMGO Institute for Health and Care Research, Department of Public and Occupational Health, Amsterdam, The Netherlands; 2 VU University Medical Center, EMGO Institute for Health and Care Research, Department of Nutrition and Dietetics, Amsterdam, The Netherlands; 3 Leiden University Medical Center, Department of Pediatrics, Leiden, The Netherlands; McGill University, Canada

## Abstract

**Background:**

It is not clear whether the association between sedentary time and cardiometabolic risk exists among obese adolescents. We examined the association between screen time (TV and computer time) and cardiometabolic risk in obese Dutch adolescents.

**Methods and Findings:**

For the current cross-sectional study, baseline data of 125 Dutch overweight and obese adolescents (12–18 years) participating in the Go4it study were included. Self-reported screen time (Activity Questionnaire for Adolescents and Adults) and clustered and individual cardiometabolic risk (i.e. body composition, systolic and diastolic blood pressure, low-density (LDL-C), high-density (HDL-C) and total cholesterol (TC), triglycerides, glucose and insulin) were assessed in all participants. Multiple linear regression analyses were used to assess the association between screen time and cardiometabolic risk, adjusting for age, gender, pubertal stage, ethnicity and moderate-to-vigorous physical activity. We found no significant relationship between self-reported total screen time and clustered cardiometabolic risk or individual risk factors in overweight and obese adolescents. Unexpectedly, self-reported computer time, but not TV time, was slightly but significantly inversely associated with TC (B = −0.002; CI = [−0.003;−0.000]) and LDL-C (B = −0.002; CI = [−0.001;0.000]).

**Conclusions:**

In obese adolescents we could not confirm the hypothesised positive association between screen time and cardiometabolic risk. Future studies should consider computer use as a separate class of screen behaviour, thereby also discriminating between active video gaming and other computer activities.

## Introduction

Paediatric obesity is an important public health issue [1]. Overweight and obese children and adolescents experience an increased cardiometabolic risk, including elevated plasma triglycerides, insulin levels and blood pressure [2]–[5]. Importantly, these metabolic risk factors are highly likely to persist in adulthood and reinforce each other, resulting in the development of type 2 diabetes and cardiovascular disease [5]–[7].

Several recent cross-sectional studies showed that screen time (i.e. TV time, computer time) was positively associated with cardiometabolic risk factors in children and adolescents [8]–[13]. To the best of our knowledge, only two studies examined this association among overweight and obese adolescents, demonstrating conflicting results [11], [14]. Pardee et al [11] found that the amount of parent-reported (children aged >8 yrs) and self-reported (children aged >8 yrs) time spent watching TV was associated with increased odds of hypertension in obese children and adolescents aged 8–17 years. In contrast, Goldfield et al [14] demonstrated that self-reported TV viewing time as well as recreational computer time were not associated with increased blood pressure or plasma lipids in overweight and obese adolescents aged 14–18 years. Thus, to date, it is unclear whether the association between screen time (TV viewing time and computer time) and cardiometabolic risk also exists in overweight and obese adolescents. Overweight and obese adolescents already have an increased cardiometabolic risk [5], [15], [16]. In order to develop effective interventions reducing cardiometabolic risk among overweight and obese adolescents, it is important to examine whether TV viewing and computer use should be discouraged in addition to stimulating physical activity. Therefore, we examined cross-sectional associations between screen time and clustered and individual cardiometabolic risk in Dutch adolescents with overweight and obesity. We hypothesised a positive association between screen time and cardiometabolic risk in overweight and obese adolescents.

## Methods

### Ethics Statement

This study was approved by the ethics committee of the VU University Medical Center and is in accordance with the Declaration of Helsinki. All participants and their parents provided written informed consent prior to their inclusion in the study.

### Design and participants

For the current cross-sectional study we used baseline data of the Go4it study. All overweight and obese adolescents in the age range of 12–18 years who were referred to the outpatient paediatric obesity clinic of the VU University Medical Center, Amsterdam, The Netherlands were invited to participate. The Go4it study is a randomised controlled trial evaluating the cost-effectiveness of a multidisciplinary group treatment for overweight and obese adolescents. Adolescents were eligible when they met the following criteria: 1) aged between 12–18 years; 2) being overweight or obese according to the International Obesity Task Force (IOTF) definition [17] and 3) Dutch speaking. Obesity as a result of a known syndrome or medical cause (hypothyroidism), mental retardation, physical limitations and diagnosed type 2 diabetes were exclusion criteria. The adolescents were measured between November 2006 and August 2008. Detailed information on the study design and methods have been reported elsewhere [18].

### Measurements

All measurements were performed by a paediatric-endocrinologist, according to a standardised protocol. Body weight (kg) and height (m) were measured in order to calculate body mass index (BMI, kg/m^2^). Trunk and total fat mass were determined using dual energy x-ray absorptiometry (DEXA). Pubertal stage was measured according to Tanner [19]. Ethnicity was assessed by self-report questioning whether one of the parents was born in a foreign country outside Europe. Systolic and diastolic blood pressure (mmHg) were measured on the non-dominant arm in supine position using an automatic device (Dinamap, Critikon, Germany), and averaging 2 measurements (with a 5 min rest interval). After an overnight fast, participants were given an Oral Glucose Tolerance Test (OGTT) in the morning (e.g. before 10am), in a dose of 1.75 g per kg of body weight (up to a maximum of 75 g), and blood samples were obtained after zero, 30 and 120 minutes. Plasma levels of glucose, insulin, triglycerides (TG), total cholesterol (TC), low-density lipoprotein cholesterol (LDL-C) and high-density lipoprotein cholesterol (HDL-C) were analyzed by enzymatic assay (Hitachi Modular P800).

The average minutes per day (weekdays and weekend days combined) spent on screen time and moderate-to-vigorous physical activity (MVPA) was assessed using the Activity Questionnaire for Adolescents & Adults (AQuAA) [20]. Adolescents tend to underestimate their sedentary time and overestimate their physical activity levels [20]. Time spent TV viewing and using the computer was assessed separately. Time spent using the computer included using the Internet and playing computer games. Screen time was calculated by summing minutes spent TV viewing and computer use. Time spent in MVPA was assessed by summing minutes spent in MVPA (i.e. active transport, leisure-time, household activities and sports).

### Statistics

Differences between genders were examined by t-tests for independent samples. A standardized continuously distributed variable (z-score) for clustered cardiometabolic risk (zMetRisk) was calculated from fasting insulin, glucose, HDL-C, TG, body fat and blood pressure. Each individual risk factor was converted to z-scores, stratified by age and gender. HDL-C was then multiplied by −1, since it is inversely related to metabolic risk. Z-scores of individual risk factors were averaged to construct the clustered cardiometabolic risk score [21]. Multiple linear regression analyses were used to assess the association between screen time and clustered cardiometabolic risk (primary outcome) and individual risk factors (secondary outcome; i.e. glucose, insulin, TG, TC, LDL-C and HDL-C). Separate analyses were performed for total screen time, computer time and TV time. All analyses were adjusted for age, gender, pubertal stage, ethnicity and MVPA.Since clustered cardiometabolic risk was calculated age and gender specific, age and gender were excluded from the analyses when clustered cardiometabolic risk was the outcome.

All statistical analyses were performed using SPSS software, version 17.0. The level of significance was set at P<0.05.

## Results

Table 1 shows the characteristics of the participants (N = 125). For three boys and one girl we had no DEXA scan and for one girl we had no blood samples. Sample sizes differed therefore slightly for the different outcome measures. Adolescents were on average 14.5 years old. According to the IOTF criteria [17] 89% of the boys and 94% of the girls were obese.

**Table 1:** Participant characteristics (Mean (SD)).

	Boys (N = 54)	Girls (N = 71)	All (N = 125)
Age, y	14.3 (1.7)	14.6 (1.7)	14.5 (1.7)
Height, cm	168.6 (11.5)	164.8 (6.6)	166.5 (9.2)*
Weight, kg	93.7 (22.3)	93.3 (14.7)	93.5 (18.3)
BMI(sds)	3.0 (0.5)	2.9 (0.4)	2.9 (0.5)*
Trunk fat mass, %	37.4 (4.7)^a^	41.3 (4.1)^b^	39.7 (4.8)*
Total fat mass, %	39.1 (4.1)^a^	42.3 (3.5)^b^	40.9 (4.1)*
Systolic BP, mm Hg	111.2 (11.4)	108.0 (10.9)	109.4 (11.2)
Diastolic BP, mm Hg	61.3 (6.6)	59.2 (7.5)	60.1 (7.2)
Fasting glucose, mmol/l	5.1 (0.3)	5.0 (0.46)^b^	5.0 (0.4)
Glucose 30, mmol/l	8.0 (1.3)	7.5 (1.3)^c^	7.7 (1.3)*
Glucose 120, mmol/l	6.0 (1.1)	6.0 (1.1)^b^	6.0 (1.1)
Fasting insulin, pmol/l	117.7 (66.1)	136.5 (67.8)^b^	128.3 (67.4)
Insuline 30, pmol/l	1275.9 (821.1)	1202.1 (579.5)^b^	1234.2 (693.0)
Insulin 120, pmol/l	580.6 (381.3)	746.7 (432.8)^b^	674.4 (417.8)*
TG, mmol/l	1.1 (0.5)	1.0 (0.6)^b^	1.1 (0.6)
TC, mmol/l	4.1 (0.7)	4.0 (0.7)^b^	4.1 (0.7)
HDL-C, mmol/l	1.1 (0.3)	1.2 (0.2)^b^	1.2 (0.2)
LDL-C, mmol/l	2.5 (0.6)	2.4 (0.6)^b^	2.4 (0.6)
zMetRisk	−0.10 (0.3)^a^	0.04 (0.5)^c^	−0.02 (0.4)
TV time, min/d	123.2 (102.5)	96.1 (89.0)	107.8 (95.7)
Computer time, min/d	98.9 (93.4)	80.3 (81.9)	88.3 (87.2)
Screen time, min/d	222.1 (164.2)	176.4 (138.9)	196.2 (151.4)

* = Significantly different between boys and girls.

a N = 51;

b N = 70;

c N = 69.

BMI, body mass index; BP, blood pressure; HDL-C, high density lipoprotein cholesterol; LDL-C low density lipoprotein cholesterol; SD, standard deviation; TC, total cholesterol; TG, triglycerides; TV, television; zMetRisk, z-score for clustered cardiometabolic risk.

According to the International Diabetes Federation (IDF) criteria [22] low HDL-C was present in 29%, high TG in 11%, high systolic blood pressure in 5%, high glucose in 8% and high diastolic blood pressure in 0% of participants. BMI, trunk fat and total percentage body fat were significantly higher among girls than boys. Boys were significantly taller than girls and had higher values of insulin at 30 and 120 min after the oral glucose challenge. Adolescents spent on average 108 min/day on TV viewing and 88 min/day using the computer.

We did not find any significant associations between total screen time (TV and computer time combined) and clustered cardiometabolic risk (B = −0.000; CI = [−0.001; 0.000]) or individual cardiometabolic risk factors (i.e. BMI, trunk and total fat mass, systolic and diastolic blood pressure and blood levels of glucose, insulin, TG, TC, HDL-C and LDL-C) (Table 2). When looking at computer time separately, we found no association with clustered metabolic risk (B = −0.001; CI = [−0.002; 0.000]) (Table 2). However, there was a significant negative association with blood levels of TC (B = −0.002 l; CI = [−0.003; −0.000]) and LDL-C (B = −0.002 l; CI = [−0.001; −0.000]). These results indicate that using the computer for 1 additional hour per day was associated with 0.02 mmol/l higher blood levels of TC and LDL-C. Computer time was not significantly associated with the other individual cardiometabolic risk factors. Analyses for TV time showed no significant associations with clustered cardiometabolic risk (B = −0.000; CI = [−0.001; 0.001]) or clustered cardiometabolic risk factors (Table 2).

**Table 2:** Associations between screen time (min/day) and cardiometabolic risk factors in obese adolescents (N = 125), adjusted for age, gender, pubertal stage, ethnicity and MVPA.

	Screen time (min/day)	Computer time (min/day)	TV time (min/day)
	B (95% CI)	B (95% CI)	B (95% CI)
zMetRisk	0.000 (−0.000; 0.001)	−0.001 (−0.001; 0.001)	0.000 (−0.000; 0.00)
BMI, kg/m^2^	0.001 (−0.005; 0.006)	0.002 (−0.007; 0.012)	0.000 (−0.009; 0.008)
Trunk fat mass, %	−0.002 (−0.007; 0.004)	0.001 (−0.008; 0.011)	−0.006 (−0.015; 0.004)
Total fat mass, %	0.000 (−0.005; 0.005)	0.002 (−0.007; 0.010)	−0.002 (−0.010; 0.006)
Systolic BP, mm Hg	−0.004 (−0.017; 0.010)	−0.001 (−0.023; 0.021)	−0.007 (−0.028; 0.013)
Diastolic BP, mm Hg	−0.004 (−0.013; 0.005)	−0.008 (−0.023; 0.007)	−0.003 (−0.017; 0.011)
Fasting glucose, mmol/l	0.000 (−0.001; 0.001)	−0.000 (−0.001; 0.000)	0.000 (0.000; 0.001)
Glucose 30, mmol/l	0.000 (−0.002; 0.002)	0.001 (−0.002; 0.004)	−0.001 (−0.003; 0.002)
Glucose 120, mmol/l	−0.001 (−0.002; 0.001)	−0.001 (−0.003; 0.001)	−0.001 (−0.003; 0.001)
Fasting insulin, pmol/l	−0.039 (−0.127; 0.049)	−0.086 (−0.232; 0.060)	−0.018 (−0.155; 0.119)
Insulin 30, pmol/l	−0.435 (−1.362; 0.491)	−1.014 (−2.549; 0.521)	−0.166 (−1.610; 1.277)
Insulin 120, pmol/l	−0.418 (−1.261; 0.425)	−0.706 (−1.603; 0.191)	−0.418 (−1.261; 0.425)
TG, mmol/l	−0.000 (−0.001; 0.001)	−0.000 (−0.002; 0.001)	0.000 (−0.001; 0.001)
TC, mmol/l	−0.001 (−0.002; 0.000)	−0.002 (−0.003; −0.000)*	0.000 (−0.002; 0.001)
HDL-C, mmol/l	−0.000 (−0.001; 0.000)	−0.000 (−0.001; 0.000)	0.000 (−0.001; 0.000)
LDL-C, mmol/l	−0.001 (−0.001; 0.000)	−0.002 (−0.001; −0.000)*	0.000 (−0.001; 0.001)

* = significantly associated with computer time (p<0.05 for TC; p<0.01 for LDL).

B, unstandardised regression coefficient; BMI, body mass index; BP, blood pressure; CI; confidence interval; HDL-C, high density lipoprotein cholesterol; LDL-C low density lipoprotein cholesterol; MVPA, moderate-to-vigorous physical activity; TG, triglycerides; TC, total cholesterol; TV, television; zMetRisk, z-score for clustered cardiometabolic risk.

## Discussion

This study showed no significant associations of total screen time and TV time with cardiometabolic risk in adolescents with overweight and obesity. Our findings are in agreement with recent findings among overweight and obese adolescents [14], but in contrast with previous findings among obese children [11] and among normal weight children and/or adolescents [9], [13], [23].

The lack of an association of screen time and TV time with cardiometabolic risk in the present study may be attributed to several factors. First, the potential association between screen time and cardiometabolic risk factors could be masked by underreporting of screen time. Obese adolescents may have underreported their actual screen time, as also has been observed for food intake [24]. Similarly, in the study of Slootmaker et al [25] it was observed that overweight adolescents overrated their physical activity level. Second, mainly obese adolescents participated in the present study. Although variances of metabolic values were comparable to previous studies [11], [23], the relatively high metabolic values could have masked a potential association between screen time and cardiometabolic risk. It is unclear why Pardee et al [11] found, in a comparable cross-sectional study and among a comparable group of children, a positive association between self-reported TV time and hypertension, whereas we found no association between TV time systolic and diastolic blood pressure.

Our finding of a small but significant negative association between computer time and blood levels of TC and LDL-C is in contrast with the recent cross-sectional study of Goldfield [14]. This study among 282 overweight and obese adolescents aged 14–18 years old found that self-reported time spent playing seated video games, but not recreational computer time, was negatively associated with cardiometabolic risk factors. Unfortunately, we could not differentiate between time spent playing video games and other computer activities, thereby limiting a detailed comparison of our findings with the findings of Goldfield [14].

In a study among 7–10 years old boys, Wang & Perry demonstrated that seated video gaming resulted in various physiological and metabolic responses, indicating that video gaming is not an entirely passive activity in children. Although our measure of computer time was broader than video gaming only, similar to Wang & Perry [26] our results could suggest that computer use may have different cardiometabolic effects than TV viewing.

Limitations of this study include the self-reported screen time and MVPA using the AQuAA. Among normal weight adolescents, the reliability of the AQuAA was fair to moderate, but this questionnaire – similar to other self-report measures – considerably underestimates sedentary time and overestimates time spent on physical activity [20]. Unfortunately, we currently do not have a gold standard for the measurement of screen time available. Accelerometry is increasingly used as an objectively measure of sedentary behaviour; however, it cannot distinguish between TV time and computer time. In addition, the cross-sectional design limits the ability to conclude on causality within the associations found. Finally, we did not have data on energy intake, cigarette smoking and alcohol consumption, which may have attenuated the associations. Strengths include the objective measures of all outcome variables, including trunk and total fat mass (determined using DEXA) and indicators of glucose and lipid metabolism (determined using fasting blood samples). In addition to fasting blood samples, this study also included an OGTT.

## Conclusions

In overweight and obese adolescents we could not confirm the hypothesised association between screen time and cardiometabolic risk. The small but significant negative association between computer time, but not TV time, and blood levels of TC and LDL-C suggest that computer time may have a differential metabolic effect than TV time. More prospective research in larger samples of overweight/obese children and adolescents on the association of TV time and computer time with cardiometabolic risk is required to confirm our findings. Moreover, long-term prospective research is needed to explore the clinical relevance of the small associations found. In these studies, researchers should also discriminate between seated and active video game playing and other computer activities.
